# Diazido­bis{2-[3-(dimethyl­amino)propyl­imino­meth­yl]phenol}manganese(III) perchlorate

**DOI:** 10.1107/S1600536808023349

**Published:** 2008-07-31

**Authors:** Gui-Bin Yang, Zhen-Hai Sun

**Affiliations:** a School of Chemistry and Life Sciences, Harbin University, Harbin 150080, People’s Republic of China

## Abstract

The title compound, [Mn(N_3_)_2_(C_12_H_18_N_2_O)_2_]ClO_4_, was synthesized from manganese(III) acetate, sodium azide and 2-[3-(dimethyl­amino)propyl­imino­meth­yl]phenol by a hydro­thermal reaction. The Mn^III^ ion is hexa­coordinated by two N and two O atoms from two phenolate ligands and two N atoms from two azide ligands. The Mn^III^ cation lies on an inversion centre and, as a result, the asymmetric unit comprises one half-mol­ecule.

## Related literature

For related literature, see: Choudhury *et al.* (2001 ▶); Church & Halvorson (1959 ▶); Chung *et al.* (1971 ▶); Okabe & Oya (2000 ▶); Serre *et al.* (2005 ▶); Scapin *et al.* (1997 ▶). 
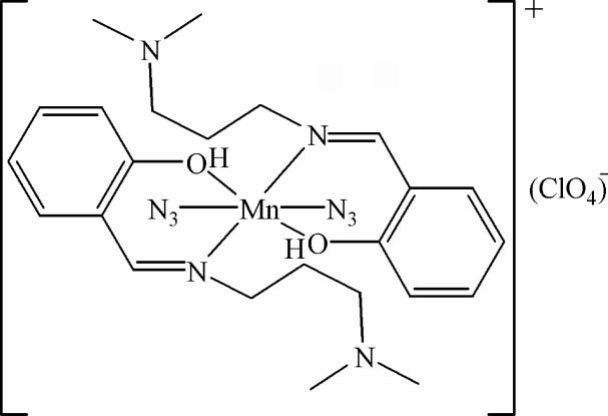


## Experimental

### 

#### Crystal data

[Mn(N_3_)_2_(C_12_H_18_N_2_O)_2_]ClO_4_
*M* *_r_* = 651.02
Monoclinic, 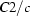

*a* = 16.8115 (17) Å
*b* = 16.4456 (18) Å
*c* = 12.9059 (14) Å
β = 121.121 (8)°
*V* = 3054.6 (6) Å^3^
*Z* = 4
Mo *K*α radiation
μ = 0.57 mm^−1^
*T* = 293 (2) K
0.43 × 0.28 × 0.22 mm

#### Data collection

Bruker APEXII CCD area-detector diffractometer
Absorption correction: multi-scan (*SADABS*; Bruker, 2001 ▶) *T*_min_ = 0.790, *T*_max_ = 0.884
3388 measured reflections
2842 independent reflections
2216 reflections with *I* > 2σ(*I*)
*R*_int_ = 0.044

#### Refinement

*R*[*F* ^2^ > 2σ(*F* ^2^)] = 0.043
*wR*(*F* ^2^) = 0.144
*S* = 1.00
2842 reflections
195 parameters
H atoms treated by a mixture of independent and constrained refinement
Δρ_max_ = 0.48 e Å^−3^
Δρ_min_ = −0.48 e Å^−3^

### 

Data collection: *APEX2* (Bruker, 2004 ▶); cell refinement: *SAINT-Plus* (Bruker, 2001 ▶); data reduction: *SAINT-Plus*; program(s) used to solve structure: *SHELXS97* (Sheldrick, 2008 ▶); program(s) used to refine structure: *SHELXL97* (Sheldrick, 2008 ▶); molecular graphics: *SHELXTL* (Sheldrick, 2008 ▶); software used to prepare material for publication: *SHELXTL*.

## Supplementary Material

Crystal structure: contains datablocks I, global. DOI: 10.1107/S1600536808023349/ez2129sup1.cif

Structure factors: contains datablocks I. DOI: 10.1107/S1600536808023349/ez2129Isup2.hkl

Additional supplementary materials:  crystallographic information; 3D view; checkCIF report

## Figures and Tables

**Fig. 1.**
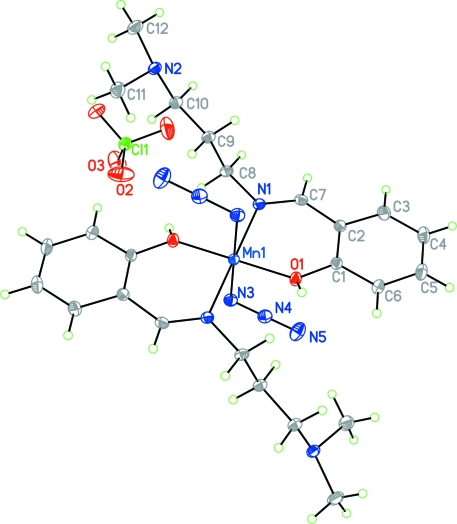
The molecular structure of (I), drawn with 30% probability displacement ellipsoids for the non-hydrogen atoms.

## supplementary crystallographic information

### Comment

In recent years, Schiff base ligands have been widely used as polydentate ligands that can coordinate to transition or rare earth ions yielding complexes with interesting properties that are useful in materials science (Church & Halvorson, 1959; Chung *et al.*, 1971) and in biological systems (Okabe & Oya, 2000; Serre *et al.*, 2005; Scapin *et al.*, 1997). Herein, we report the synthesis and X-ray crystal structure analysis of the title compound, (I).

The molecular structure of (I) is shown in Fig. 1. The Mn^III^ cation lies on an inversion centre, as a consequence the asymmetric unit comprises half of the molecule. The Mn^III^ ion is hexacoordinated by two N and two O atoms from two 2-[3-(dimethylamino)propyliminomethyl]phenolate ligands and two N atoms from two azide ligands.

### Experimental

The title compound was synthesized according to the following two steps:

(i) Synthesis of the ligand: 2-[3-(dimethylamino)propyliminomethyl]phenol was prepared by refluxing 3-dimethylamino-1-propylamine (1.0 mmol) and salicylaldehyde (1.0 mmol) in ethanol (25 ml) for two hours and used without further purification, according to the literature method (see: Choudhury *et al.*, 2001).

(ii) Synthesis of the complex: A solution of sodium azide (0.5 mmol) and sodium perchlorate (0.05 mmol) in 5 ml water was added to the ethanol solution of the ligand (1.0 mmol). Then manganese(III) acetate dihydrate (0.5 mmol) in 3 ml water was added to the above mixture. A yellow mixture was obtained by refluxing for 3 h and was left to stand undisturbed. Upon slow evaporation at room temperature, light yellow prismatic crystals suitable for X-ray diffraction appeared three days later and were separated by filtration.

### Refinement

The H atom on O1 was located from a difference density map and was refined with a distance restraint of d(O—H) = 0.82 (2) Å. All other H atoms were placed in calculated positions with C—H = 0.93 Å and N—H = 0.86 Å and refined as riding with *U*_iso_(H) = 1.2*U*_eq_(carrier).

### Crystal data

**Table:**

[Mn(N_3_)_2_(C_12_H_18_N_2_O)_2_]ClO_4_	*F*_000_ = 1360
*M**_r_* = 651.02	*D*_x_ = 1.416 Mg m^−^^3^
Monoclinic, *C*2/*c*	Mo *K*α radiation λ = 0.71073 Å
Hall symbol: -C 2yc	Cell parameters from 2842 reflections
*a* = 16.8115 (17) Å	θ = 1.9–25.5º
*b* = 16.4456 (18) Å	µ = 0.58 mm^−^^1^
*c* = 12.9059 (14) Å	*T* = 293 (2) K
β = 121.121 (8)º	Prism, yellow
*V* = 3054.6 (6) Å^3^	0.43 × 0.28 × 0.22 mm
*Z* = 4	

### Data collection

**Table:**

Bruker APEXII CCD area-detector diffractometer	2842 independent reflections
Radiation source: fine-focus sealed tube	2216 reflections with *I* > 2σ(*I*)
Monochromator: graphite	*R*_int_ = 0.044
*T* = 293(2) K	θ_max_ = 25.5º
φ and ω scans	θ_min_ = 1.9º
Absorption correction: multi-scan(SADABS; Bruker, 2001)	*h* = −20→1
*T*_min_ = 0.790, *T*_max_ = 0.884	*k* = −1→19
3388 measured reflections	*l* = −13→15

### Refinement

**Table:**

Refinement on *F*^2^	Secondary atom site location: difference Fourier map
Least-squares matrix: full	Hydrogen site location: inferred from neighbouring sites
*R*[*F*^2^ > 2σ(*F*^2^)] = 0.044	H atoms treated by a mixture of independent and constrained refinement
*wR*(*F*^2^) = 0.144	*w* = 1/[σ^2^(*F*_o_^2^) + (0.0843*P*)^2^ + 2.1116*P*] where *P* = (*F*_o_^2^ + 2*F*_c_^2^)/3
*S* = 1.00	(Δ/σ)_max_ < 0.001
2842 reflections	Δρ_max_ = 0.48 e Å^−^^3^
195 parameters	Δρ_min_ = −0.48 e Å^−^^3^
Primary atom site location: structure-invariant direct methods	Extinction correction: none

### Special details

**Table:**

Geometry. All e.s.d.'s (except the e.s.d. in the dihedral angle between two l.s. planes) are estimated using the full covariance matrix. The cell e.s.d.'s are taken into account individually in the estimation of e.s.d.'s in distances, angles and torsion angles; correlations between e.s.d.'s in cell parameters are only used when they are defined by crystal symmetry. An approximate (isotropic) treatment of cell e.s.d.'s is used for estimating e.s.d.'s involving l.s. planes.
Refinement. Refinement of *F*^2^ against ALL reflections. The weighted *R*-factor *wR* and goodness of fit *S* are based on *F*^2^, conventional *R*-factors *R* are based on *F*, with *F* set to zero for negative *F*^2^. The threshold expression of *F*^2^ > σ(*F*^2^) is used only for calculating *R*-factors(gt) *etc*. and is not relevant to the choice of reflections for refinement. *R*-factors based on *F*^2^ are statistically about twice as large as those based on *F*, and *R*- factors based on ALL data will be even larger.

### Fractional atomic coordinates and isotropic or equivalent isotropic displacement parameters (Å^2^)

**Table:**

	*x*	*y*	*z*	*U*_iso_*/*U*_eq_	
Mn1	0.2500	0.2500	0.5000	0.0467 (2)	
Cl1	0.0000	0.15323 (8)	0.7500	0.0711 (3)	
O1	0.28801 (14)	0.20397 (12)	0.40192 (19)	0.0626 (5)	
O2	0.0690 (2)	0.2006 (3)	0.7580 (4)	0.1579 (17)	
O3	0.0340 (3)	0.1044 (2)	0.8529 (3)	0.1482 (15)	
N1	0.13856 (14)	0.16698 (13)	0.43275 (19)	0.0481 (5)	
N2	−0.12645 (16)	0.32706 (14)	0.3901 (2)	0.0576 (6)	
N3	0.33339 (17)	0.16169 (15)	0.6490 (2)	0.0603 (6)	
N4	0.36786 (18)	0.10586 (17)	0.6266 (2)	0.0649 (6)	
N5	0.4004 (3)	0.0528 (2)	0.6034 (4)	0.0980 (10)	
C1	0.25785 (18)	0.14208 (15)	0.3267 (2)	0.0497 (6)	
C2	0.18274 (18)	0.09312 (16)	0.3069 (2)	0.0523 (6)	
C3	0.1561 (2)	0.0279 (2)	0.2252 (3)	0.0730 (9)	
H3A	0.1071	−0.0055	0.2123	0.088*	
C4	0.2015 (3)	0.0129 (2)	0.1639 (4)	0.0896 (11)	
H4A	0.1828	−0.0300	0.1092	0.108*	
C5	0.2746 (3)	0.0614 (2)	0.1835 (3)	0.0791 (10)	
H5A	0.3050	0.0512	0.1417	0.095*	
C6	0.3033 (2)	0.12473 (19)	0.2640 (3)	0.0633 (7)	
H6A	0.3534	0.1565	0.2771	0.076*	
C7	0.12924 (18)	0.10820 (16)	0.3631 (2)	0.0513 (6)	
H7A	0.0826	0.0708	0.3467	0.062*	
C8	0.06994 (18)	0.17367 (16)	0.4719 (3)	0.0532 (6)	
H8A	0.1022	0.1806	0.5590	0.064*	
H8B	0.0336	0.1241	0.4513	0.064*	
C9	0.0061 (2)	0.24542 (17)	0.4104 (3)	0.0572 (7)	
H9A	−0.0247	0.2389	0.3233	0.069*	
H9B	0.0427	0.2949	0.4322	0.069*	
C10	−0.0665 (2)	0.25332 (17)	0.4460 (3)	0.0583 (7)	
H10A	−0.1053	0.2051	0.4203	0.070*	
H10B	−0.0358	0.2570	0.5335	0.070*	
C11	−0.0775 (3)	0.4036 (2)	0.4465 (4)	0.0850 (10)	
H11A	−0.0216	0.4066	0.4438	0.127*	
H11B	−0.0621	0.4052	0.5292	0.127*	
H11C	−0.1169	0.4488	0.4033	0.127*	
C12	−0.2131 (2)	0.3213 (2)	0.3943 (4)	0.0844 (11)	
H12A	−0.2501	0.3691	0.3585	0.127*	
H12B	−0.1974	0.3170	0.4770	0.127*	
H12C	−0.2477	0.2742	0.3502	0.127*	
H1A	0.313 (2)	0.2343 (11)	0.376 (3)	0.080*	

### Atomic displacement parameters (Å^2^)

**Table:**

	*U*^11^	*U*^22^	*U*^33^	*U*^12^	*U*^13^	*U*^23^
Mn1	0.0433 (3)	0.0501 (3)	0.0527 (3)	−0.0081 (2)	0.0290 (3)	−0.0109 (2)
Cl1	0.0692 (7)	0.0852 (8)	0.0727 (7)	0.000	0.0465 (6)	0.000
O1	0.0654 (12)	0.0654 (12)	0.0774 (13)	−0.0213 (10)	0.0513 (11)	−0.0260 (10)
O2	0.083 (2)	0.220 (4)	0.168 (4)	−0.032 (3)	0.062 (2)	0.055 (3)
O3	0.219 (4)	0.128 (3)	0.087 (2)	−0.018 (3)	0.071 (2)	0.0178 (19)
N1	0.0422 (11)	0.0487 (12)	0.0532 (12)	−0.0003 (9)	0.0244 (9)	0.0002 (10)
N2	0.0499 (12)	0.0601 (14)	0.0690 (14)	0.0035 (11)	0.0352 (11)	−0.0025 (11)
N3	0.0555 (13)	0.0637 (15)	0.0616 (14)	−0.0029 (12)	0.0301 (12)	0.0023 (12)
N4	0.0647 (15)	0.0638 (16)	0.0727 (16)	−0.0071 (13)	0.0401 (14)	0.0059 (13)
N5	0.127 (3)	0.0709 (19)	0.135 (3)	0.0137 (19)	0.096 (3)	0.0101 (19)
C1	0.0521 (14)	0.0471 (13)	0.0497 (14)	0.0050 (11)	0.0262 (12)	−0.0020 (11)
C2	0.0484 (14)	0.0480 (14)	0.0532 (14)	0.0048 (11)	0.0212 (12)	−0.0027 (11)
C3	0.073 (2)	0.0593 (17)	0.079 (2)	−0.0060 (15)	0.0346 (17)	−0.0179 (16)
C4	0.102 (3)	0.079 (2)	0.094 (3)	−0.008 (2)	0.055 (2)	−0.039 (2)
C5	0.095 (3)	0.080 (2)	0.079 (2)	0.006 (2)	0.057 (2)	−0.0181 (18)
C6	0.0683 (18)	0.0650 (17)	0.0686 (18)	0.0052 (15)	0.0438 (15)	−0.0039 (14)
C7	0.0425 (13)	0.0456 (14)	0.0567 (15)	−0.0028 (11)	0.0190 (11)	0.0005 (12)
C8	0.0428 (13)	0.0569 (15)	0.0642 (16)	−0.0031 (12)	0.0307 (12)	0.0028 (13)
C9	0.0483 (15)	0.0666 (18)	0.0628 (17)	0.0030 (13)	0.0329 (13)	0.0054 (13)
C10	0.0521 (16)	0.0656 (18)	0.0652 (17)	0.0022 (13)	0.0359 (14)	0.0042 (13)
C11	0.082 (2)	0.068 (2)	0.114 (3)	−0.0071 (18)	0.056 (2)	−0.019 (2)
C12	0.0615 (19)	0.089 (2)	0.119 (3)	0.0021 (18)	0.059 (2)	−0.008 (2)

### Geometric parameters (Å, °)

**Table:**

Mn1—O1	1.8493 (18)	C3—C4	1.377 (5)
Mn1—O1^i^	1.8493 (18)	C3—H3A	0.9300
Mn1—N1^i^	2.109 (2)	C4—C5	1.374 (5)
Mn1—N1	2.109 (2)	C4—H4A	0.9300
Mn1—N3^i^	2.233 (2)	C5—C6	1.370 (4)
Mn1—N3	2.233 (2)	C5—H5A	0.9300
Cl1—O2	1.357 (3)	C6—H6A	0.9300
Cl1—O2^ii^	1.357 (3)	C7—H7A	0.9300
Cl1—O3^ii^	1.397 (3)	C8—C9	1.515 (4)
Cl1—O3	1.397 (3)	C8—H8A	0.9700
O1—C1	1.314 (3)	C8—H8B	0.9700
O1—H1A	0.828 (9)	C9—C10	1.516 (4)
N1—C7	1.273 (3)	C9—H9A	0.9700
N1—C8	1.483 (3)	C9—H9B	0.9700
N2—C11	1.474 (4)	C10—H10A	0.9700
N2—C12	1.489 (4)	C10—H10B	0.9700
N2—C10	1.502 (4)	C11—H11A	0.9600
N3—N4	1.199 (4)	C11—H11B	0.9600
N4—N5	1.149 (4)	C11—H11C	0.9600
C1—C6	1.400 (4)	C12—H12A	0.9600
C1—C2	1.405 (4)	C12—H12B	0.9600
C2—C3	1.405 (4)	C12—H12C	0.9600
C2—C7	1.439 (4)		
			
O1—Mn1—O1^i^	180.00 (8)	C5—C4—H4A	120.0
O1—Mn1—N1^i^	89.94 (8)	C3—C4—H4A	120.0
O1^i^—Mn1—N1^i^	90.06 (8)	C6—C5—C4	120.8 (3)
O1—Mn1—N1	90.06 (8)	C6—C5—H5A	119.6
O1^i^—Mn1—N1	89.94 (8)	C4—C5—H5A	119.6
N1^i^—Mn1—N1	180.00 (13)	C5—C6—C1	120.7 (3)
O1—Mn1—N3^i^	87.82 (10)	C5—C6—H6A	119.7
O1^i^—Mn1—N3^i^	92.18 (10)	C1—C6—H6A	119.7
N1^i^—Mn1—N3^i^	87.83 (8)	N1—C7—C2	127.3 (2)
N1—Mn1—N3^i^	92.17 (8)	N1—C7—H7A	116.4
O1—Mn1—N3	92.18 (10)	C2—C7—H7A	116.4
O1^i^—Mn1—N3	87.82 (10)	N1—C8—C9	110.2 (2)
N1^i^—Mn1—N3	92.17 (8)	N1—C8—H8A	109.6
N1—Mn1—N3	87.83 (8)	C9—C8—H8A	109.6
N3^i^—Mn1—N3	180.0	N1—C8—H8B	109.6
O2—Cl1—O2^ii^	109.9 (5)	C9—C8—H8B	109.6
O2—Cl1—O3^ii^	108.4 (3)	H8A—C8—H8B	108.1
O2^ii^—Cl1—O3^ii^	110.1 (2)	C8—C9—C10	111.8 (2)
O2—Cl1—O3	110.1 (2)	C8—C9—H9A	109.2
O2^ii^—Cl1—O3	108.4 (3)	C10—C9—H9A	109.2
O3^ii^—Cl1—O3	109.8 (3)	C8—C9—H9B	109.3
C1—O1—Mn1	133.21 (18)	C10—C9—H9B	109.3
C1—O1—H1A	104.7 (14)	H9A—C9—H9B	107.9
Mn1—O1—H1A	117.3 (13)	N2—C10—C9	111.7 (2)
C7—N1—C8	117.6 (2)	N2—C10—H10A	109.3
C7—N1—Mn1	122.76 (18)	C9—C10—H10A	109.3
C8—N1—Mn1	119.59 (17)	N2—C10—H10B	109.3
C11—N2—C12	110.1 (3)	C9—C10—H10B	109.3
C11—N2—C10	112.8 (2)	H10A—C10—H10B	107.9
C12—N2—C10	111.0 (3)	N2—C11—H11A	109.5
N4—N3—Mn1	117.2 (2)	N2—C11—H11B	109.5
N5—N4—N3	179.0 (3)	H11A—C11—H11B	109.5
O1—C1—C6	117.9 (3)	N2—C11—H11C	109.5
O1—C1—C2	123.1 (2)	H11A—C11—H11C	109.5
C6—C1—C2	119.0 (3)	H11B—C11—H11C	109.5
C1—C2—C3	118.8 (3)	N2—C12—H12A	109.5
C1—C2—C7	123.1 (2)	N2—C12—H12B	109.5
C3—C2—C7	118.0 (3)	H12A—C12—H12B	109.5
C4—C3—C2	120.7 (3)	N2—C12—H12C	109.5
C4—C3—H3A	119.6	H12A—C12—H12C	109.5
C2—C3—H3A	119.6	H12B—C12—H12C	109.5
C5—C4—C3	119.9 (3)		

Symmetry codes: (i) −*x*+1/2, −*y*+1/2, −*z*+1; (ii) −*x*, *y*, −*z*+3/2.
